# Bone Marrow-Mesenchymal Stromal Cell Secretome as Conditioned Medium Relieves Experimental Skeletal Muscle Damage Induced by Ex Vivo Eccentric Contraction

**DOI:** 10.3390/ijms22073645

**Published:** 2021-03-31

**Authors:** Roberta Squecco, Alessia Tani, Flaminia Chellini, Rachele Garella, Eglantina Idrizaj, Irene Rosa, Sandra Zecchi-Orlandini, Mirko Manetti, Chiara Sassoli

**Affiliations:** 1 Section of Physiological Sciences, Department of Experimental and Clinical Medicine, University of Florence, 50134 Florence, Italy; roberta.squecco@unifi.it (R.S.); rachele.garella@unifi.it (R.G.); eglantina.idrizaj@unifi.it (E.I.); 2 Section of Anatomy and Histology, Department of Experimental and Clinical Medicine, University of Florence, 50134 Florence, Italy; alessia.tani@unifi.it (A.T.); flaminia.chellini@unifi.it (F.C.); irene.rosa@unifi.it (I.R.); sandra.zecchi@unifi.it (S.Z.-O.)

**Keywords:** biophysical properties, eccentric contraction, mesenchymal stem/stromal cell (MSC), repair/regeneration, satellite cells, secretome, skeletal muscle, stem cell, telocytes/CD34^+^ stromal cells

## Abstract

Bone marrow-mesenchymal stem/stromal cells (MSCs) may offer promise for skeletal muscle repair/regeneration. Growing evidence suggests that the mechanisms underpinning the beneficial effects of such cells in muscle tissue reside in their ability to secrete bioactive molecules (secretome) with multiple actions. Hence, we examined the effects of MSC secretome as conditioned medium (MSC-CM) on ex vivo murine extensor digitorum longus muscle injured by forced eccentric contraction (EC). By combining morphological (light and confocal laser scanning microscopies) and electrophysiological analyses we demonstrated the capability of MSC-CM to attenuate EC-induced tissue structural damages and sarcolemnic functional properties’ modifications. MSC-CM was effective in protecting myofibers from apoptosis, as suggested by a reduced expression of pro-apoptotic markers, cytochrome c and activated caspase-3, along with an increase in the expression of pro-survival AKT factor. Notably, MSC-CM also reduced the EC-induced tissue redistribution and extension of telocytes/CD34^+^ stromal cells, distinctive cells proposed to play a “nursing” role for the muscle resident myogenic satellite cells (SCs), regarded as the main players of regeneration. Moreover, it affected SC functionality likely contributing to replenishment of the SC reservoir. This study provides the necessary groundwork for further investigation of the effects of MSC secretome in the setting of skeletal muscle injury and regenerative medicine.

## 1. Introduction

Skeletal muscle injuries represent the most frequent and debilitating damages in sport activities, accounting for 10 to 55% of all acute sport injuries depending on the sport type, as well as in recreation physical activities, worldwide. They usually occur after a direct or indirect insult. In the direct trauma, there is an external force impacting on the muscle, causing contusion or laceration, and hence, tissue damage. In contrast, in the indirect trauma, when no traumatic forces are applied to the muscle, the lesion is mainly dependent by an eccentric contraction (EC) of the muscle [1,2].

About 35–55% of sport injuries cause myofiber structural damage [3]. Other leading causes of skeletal muscle damage/dysfunction are tissue wasting diseases such as cachexia, sarcopenia and muscular dystrophies. Furthermore, it is worth mentioning that myopathies predisposing to muscle damage are complications related to some complex systemic disorders with an increasing incidence worldwide, such as diabetes [2,4,5].

The adult skeletal muscle possesses the capability to regenerate lost tissue upon injury mainly thanks to the activity of a small population of muscle tissue resident stem cells. These are located at the periphery of adult myofibers, between the sarcolemma and the surrounding basal lamina, thus being referred to as satellite cells (SCs). Their activity is strictly regulated by several factors acting within their specialized niche, namely bioactive molecules, different cell types and the surrounding extracellular matrix [6,7]. In this context, telocytes/CD34^+^ stromal cells, a distinctive type of interstitial cell recently identified in the skeletal muscle stroma, may serve as key “nursing” cells supporting SC activation [8]. Nevertheless, extensive or severe damage with an intense and/or persistent inflammatory reaction or a pathological microenvironment, overwhelms the innate muscle tissue regenerative capacity, ultimately resulting in the formation of non-contractile fibrotic scar tissue, replacing the damaged myofibers [9,10]. Muscle injuries should not be underestimated, since an improper treatment determines either significant undesirable short-term or long-term sequelae including time lost from work, increase of re-injury risk, inability to fully recover pre-injury performances and, in the worst case, a permanent functional debilitation, with high socio-economic impact and Health Care System costs. Unfortunately, despite progresses, mostly in the employed technologies [11], conventional conservative pharmacological or physical-rehabilitative therapies are not always satisfactory and fully effective in achieving the complete morpho-functional recovery of damaged muscle tissue. Surgical approach and muscle flap/graft transplantation remain the leading options for severe damage with volumetric muscle loss or long-term chronic pain with obvious complications and limitations mainly related to the tissue availability [3]. In addition, there is no clear consensus on the best treatment to manage muscle lesions [2]. Therefore, the identification of alternative therapeutic strategies able to prevent the aberrant fibrotic reparative response and to improve the endogenous tissue regenerative mechanisms represents an urgent clinical need. Indeed, this is the requisite to preserve and/or recreate the native tissue structure that guarantees muscle functionality.

In such a context, bone marrow-mesenchymal stem/stromal cells (MSCs) endowed with regenerative capacities, immune-privileged and anti-inflammatory properties and, in theory, with no practical and ethical restrictions [12], may be highly promising [13,14,15,16,17,18,19,20,21]. Growing evidence suggests that the mechanisms underpinning the beneficial effects and regenerative capabilities of MSCs in skeletal muscle tissue reside in their ability to secrete a broad range of bioactive molecules rather than in their inherent multipotency. These biomolecules are produced in the form of either soluble factors or are packaged within extracellular vesicles (exosomes and microvesicles) containing growth factors, cytokines, mRNAs, and miRNAs, and are collectively referred to as secretome endowed with pro-regenerative effects on the host microenvironment [22,23,24,25,26,27,28,29,30]. The quite rapid disappearance of the transplanted MSCs, observed concomitantly to the favorable outcomes in terms of morpho-functional recovery of damaged muscle, is a further argument in favor of a paracrine action of these cells [18,28]. However, the actual capability of MSCs to aid the healing of injured skeletal muscle is still controversial [31,32]. Moreover, the effects of the MSC paracrine factors and the underlying cellular and molecular mechanisms are worth further investigation.

In the present study, we aimed to investigate the effects of murine MSC secretome, as conditioned medium (CM), in a murine model of ex vivo extensor digitorum longus (EDL) muscle damage induced by forced EC in isometric condition. We have revealed the capability of MSC-CM to attenuate EC-induced tissue structural damages and sarcolemnic functional properties’ modifications, to protect myofibers from apoptosis and to influence the behavior of the cells involved in muscle repair/regeneration, namely SCs and telocytes.

## 2. Results

### 2.1. MSC-CM Attenuates the EC-Induced Skeletal Muscle Tissue Structural Damage and Sarcolemnic Functional Properties’ Modifications

**Figure 1:**
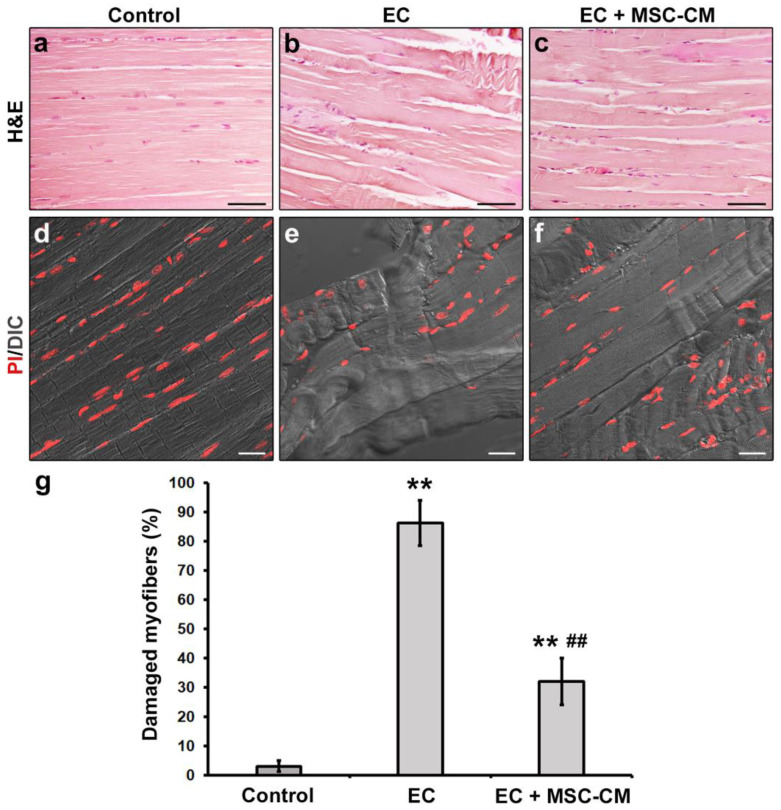
Morphological analyses of formalin-fixed and paraffin-embedded tissue sections from control murine EDL muscles, EDL muscles damaged by ex vivo forced eccentric contraction (EC) and EDL muscles damaged by EC in the presence of bone marrow-mesenchymal stromal cell (MSC) conditioned medium (EC + MSC-CM). (**a**–**c**) Representative light microscopic images of longitudinal muscle tissue sections stained with hematoxylin and eosin (H&E). Scale bars = 50 μm. (**d**–**f**) Representative superimposed confocal fluorescence and differential interference contrast (DIC, grey) images of muscle tissue sections showing nuclei stained with propidium iodide (PI, red). Scale bars = 25 μm. (**g**) Quantitative analysis of the percentage of damaged myofibers. Values are mean ± SEM. ** *p* < 0.01 vs. control, ## *p* < 0.01 vs. EC.

The successful construction of the ex vivo EC-induced EDL skeletal muscle damage model was verified by means of both morphological and electrophysiological analyses. The results were essentially in accordance with our previous reports [8,33]. Light microscopy evaluation of hematoxylin and eosin (H&E)-stained longitudinal sections of EDL muscles subjected to EC revealed that the majority of myofibers exhibited clear signs of altered morphology with respect to control muscles (*p* < 0.01, Figure 1a,b,g).

In particular, EC damaged samples exhibited myofiber disorganization and misalignment associated with morphological variability (i.e., some myofibers appeared swollen and/or rippled) and structural damages consisting in Z-disc smearing and streaming and focal myofilament loss. Nuclei of EC myofibers appeared more rounded and distributed through the sarcoplasm in comparison to nuclei of control myofibers that, instead, appeared elongated and peripherally located just beneath the sarcolemma. Combined differential interference contrast (DIC) and fluorescence images of muscle sections labelled with propidium iodide (PI) to reveal nuclei, acquired simultaneously by confocal laser scanning microscopy, confirmed the myofiber structural alterations induced by EC (Figure 1d,e).

**Figure 2:**
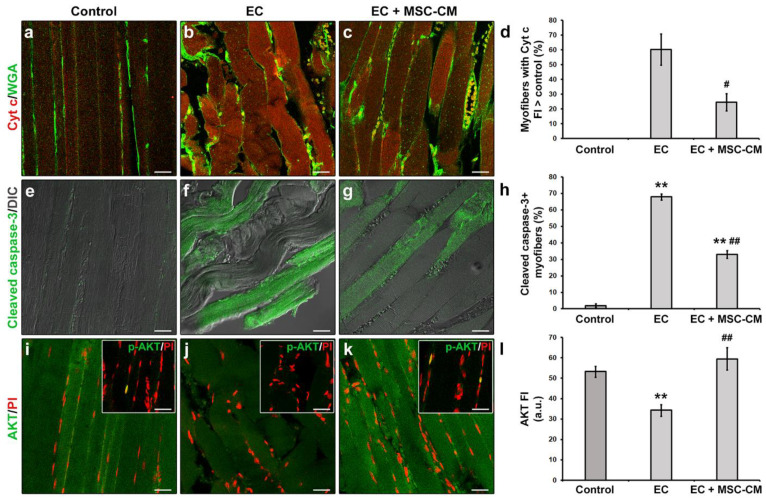
Morphological analyses of formalin-fixed and paraffin-embedded tissue sections from control EDL muscles, EDL muscles damaged by ex vivo forced eccentric contraction (EC) and EDL muscles damaged by EC in the presence of bone marrow-mesenchymal stromal cell (MSC) conditioned medium (EC + MSC-CM). (**a**–**c**) Representative confocal immunofluorescence images of muscle tissue sections immunostained with antibodies against cytochrome (Cyt) c (red) and counterstained with the membrane dye wheat germ agglutinin (WGA) (green). (**d**) Quantitative analysis of the percentage of myofibers with Cyt c fluorescence intensity (FI) higher than that of control. (**e**–**g**) Representative superimposed confocal immunofluorescence and differential interference contrast (DIC, grey) images of muscle tissue sections immunostained with antibodies against the pro-apoptotic marker activated/cleaved caspase-3 (green). (**h**) Quantitative analysis of the percentage of cleaved caspase-3^+^ myofibers. (**i**–**k**) Representative confocal immunofluorescence images of muscle tissue sections immunostained with antibodies against the pro-survival/anti-apoptotic factor AKT (green) and counterstained with the nuclear dye propidium iodide (PI, red). Insets: representative confocal immunofluorescence images of muscle tissue sections immunostained with antibodies against the activated phosphorylated form of AKT (p-AKT, green) and counterstained with PI (red). Images are representative of at least 3 sections from each of 4 control, 4 EC and 4 EC + MSC-CM muscles. Scale bars = 25 μm. (**l**) Quantitative analysis of AKT FI. a.u.: arbitrary units. Values are mean ± SEM. ** *p* < 0.01 vs. control; # *p* < 0.05 and ## *p* < 0.01 vs. EC.

Moreover, in EC-damaged muscle fibers we revealed the activation of the mitochondrial caspase-dependent apoptotic pathway. Indeed, in EC samples approximately 60% of the myofibers exhibited cytoplasmic expression levels of pro-apoptotic cytochrome c (Cyt c) higher than those of control myofibers suggestive of a release of this protein from mitochondria (Figure 2a,b,d).

Moreover, a significant increase in the percentage of myofibers expressing the activated/cleaved caspase-3 was observed in EC muscles with respect to control ones (*p* < 0.01, Figure 1e,f,h). In parallel, EC-damaged myofibers exhibited a reduced expression of the pro-survival/anti-apoptotic factor AKT [34] as compared to control (*p* < 0.01, Figure 2i,j,l). Nuclear positivity for the activated phosphorylated form of AKT (p-AKT) was rarely detected in myofibers of control muscles and completely undetectable in those of EC-damaged muscles (Figure 2i,j insets).

**Figure 3:**
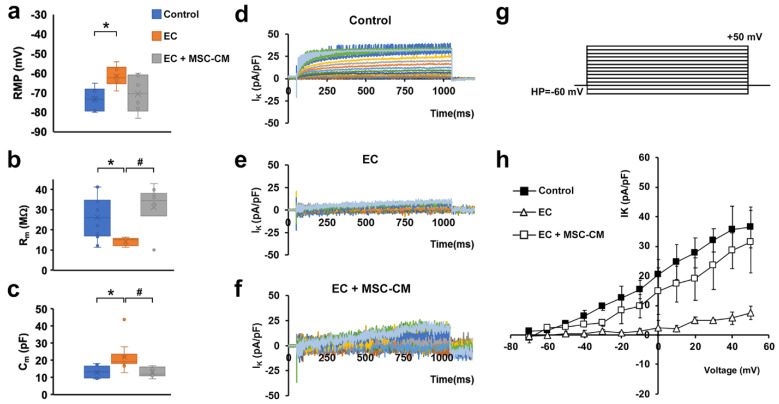
Membrane passive properties and K^+^ currents of single myofibers from control EDL muscles, EDL muscles damaged by ex vivo forced eccentric contraction (EC) and EDL muscles damaged by EC in the presence of bone marrow-mesenchymal stromal cell (MSC) conditioned medium (EC + MSC-CM). (**a**–**c**) Membrane passive properties. (**a**) Resting membrane potential (RMP) values (in mV) resulted −73.2 ± 5.7 in control myofibers (*n* = 6), −61.5 ± 5.1 in EC myofibers (*n* = 6) and −70.5 ± 9.5 in EC + MSC-CM myofibers (*n* = 6). One-way ANOVA with Bonferroni’s correction gave *p* = 0.03; F = 4.46 > Fcrit = 3.68; df = 17. (**b**) Membrane resistance (R_m_) values (in MΩ) were 26.34 ± 10.6 in control myofibers (*n* = 12), 14.3 ± 2.0 in EC myofibers (*n* = 5) and 31.3 ± 11.6 in EC + MSC-CM myofibers (*n* = 10). One-way ANOVA with Bonferroni’s correction gave *p* = 0.018 F 4.7 > Fcrit = 3.4; df = 26. (**c**) Membrane capacitance (C_m_) values (in pF) were 13.28 ± 3.3 in control myofibers (*n* = 7), 22.1 ± 8.5 in EC myofibers (*n* = 10) and 13.1 ± 2.7 in EC + MSC-CM myofibers (*n* = 9). One-way ANOVA with Bonferroni’s correction gave *p* = 0.017; F = 4.8 > Fcrit=3.4; df = 25. Values are mean ± SD; * *p* < 0.05 vs. control; # *p* < 0.05 vs. EC. N is the number of myofibers included in the statistical analysis. (**d–g**) Time course of representative K^+^ currents (I_K_) evoked in response to the voltage pulse protocol depicted in (**g**) obtained from a typical (**d**) control, (**e**) EC and (**f**) EC + MSC-CM myofiber. Current amplitudes are normalized to cell capacitance and are reported in pA/pF. (**h**) I/V plot of the overall current values in control (*n* = 7), EC (*n* = 6) and EC + MSC-CM (*n* = 7). All of the data from EC myofibers are statistically different from control starting for voltage values more positive than −40 mV. Data from EC + MSC-CM myofibers are significantly different from control ones for −30 mV values, and to EC myofibers for voltage values more positive than −20 mV. The error bar is visible when its size exceeds that of the symbol. (Two-way ANOVA, statistically significance for *p* < 0.05, not indicated in this panel for clarity).

Electrophysiological analysis performed on single myofibers showed that EC caused the occurrence of significant alterations in the functional properties of sarcolemma and, therefore, in the myofiber excitability. In particular, myofibers subjected to EC showed statistically significant differences compared to control, such as: (i) a stably maintained resting membrane potential (RMP) depolarization (Figure 3a); (ii), a decrease in membrane resistance (R_m_) value (Figure 3b), indicative of a major membrane permeability; and iii) an increase in membrane capacitance (C_m_) value (Figure 3c).

The latter parameter is likely well-correlated with the disorganization of T-tubular membrane that can determine a sarcolemnic surface increase, consistent with the aberrant morphology observed for EC myofibers.

The analysis of the ion currents elicited in the muscle fibers revealed a significantly reduced voltage-dependent K^+^ current (I_K_) amplitude in EC-damaged myofibers compared to control myofibers (Figure 3d,e) that may lead to an altered myofiber excitability. The ion currents were elicited by the pulse protocol shown in Figure 3g. This effect can be clearly observed both from the time course evaluation and from the I/V plot analysis, where the plot related to EC myofibers (Figure 3h, open triangles) exhibits a significantly smaller amplitude (evaluated at the end of the voltage step) and a different voltage dependence with respect to that referred to the control condition (Figure 3h, filled squares).

Notably, MSC-CM was able to strongly attenuate the observed morphological and electrophysiological myofiber alterations induced by EC damage. In fact, muscles undergoing EC in the presence of MSC-CM showed a significant reduction in the percentage of damaged myofibers and a reduction in damage severity as compared to EC alone (*p* < 0.01, Figure 1c,f,g). In these samples, the percentage of myofibers that showed an enhanced expression of Cyt c with respect to control decreased as compared to EC muscles (*p* < 0.05, Figure 2c,d). A reduction was observed also for the percentage of myofibers positive for cleaved caspase-3 (*p* < 0.01, Figure 2g,h). Of note, EC + MSC-CM-treated myofibers also exhibited a significant increased expression of AKT as compared to myofibers subjected to EC alone (*p* < 0.01, Figure 2k,l), with values comparable to those of the control (*p* = 0.53). At variance with EC and similarly to control muscles, sporadic p-AKT^+^ nuclei were observed in EC + MSC-CM myofibers (Figure 2i,j,k insets).

In line with the outcomes obtained by the morphological analysis, the myofibers from EC + MSC-CM muscles showed passive properties similar to control and significantly different to those recorded from EC ones (Figure 3a–c). Therefore, these results suggest a clear protective action of the MSC-CM against the membrane damage. As far as I_K_ analysis is concerned, in EC + MSC-CM myofibers the current amplitude was similar to control despite a quite different time course (Figure 3f). However, in this condition, I_K_ current was negligible for voltage steps more negative than −20 mV (Figure 3h, open squares), similar to that recorded in EC damaged myofibers (Figure 3h, open triangles). Outstandingly, it regained values not statistically different to those recorded in control conditions for voltage steps more positive than −20 mV (Figure 3h, open and filled squares). This indicates that the alteration of K^+^ channel activation and, in turn, the likely correlated myofiber excitability were not completely reverted by MSC-CM treatment.

### 2.2. MSC-CM Reduces the EC-Induced Tissue Redistribution and Extension of Telocytes/CD34^+^ Stromal Cells and Affects SC Functionality

Finally, muscle samples were analyzed by confocal microscopy for detection of the cells involved in muscle repair/regeneration, such as telocytes and SCs [8].

**Figure 4:**
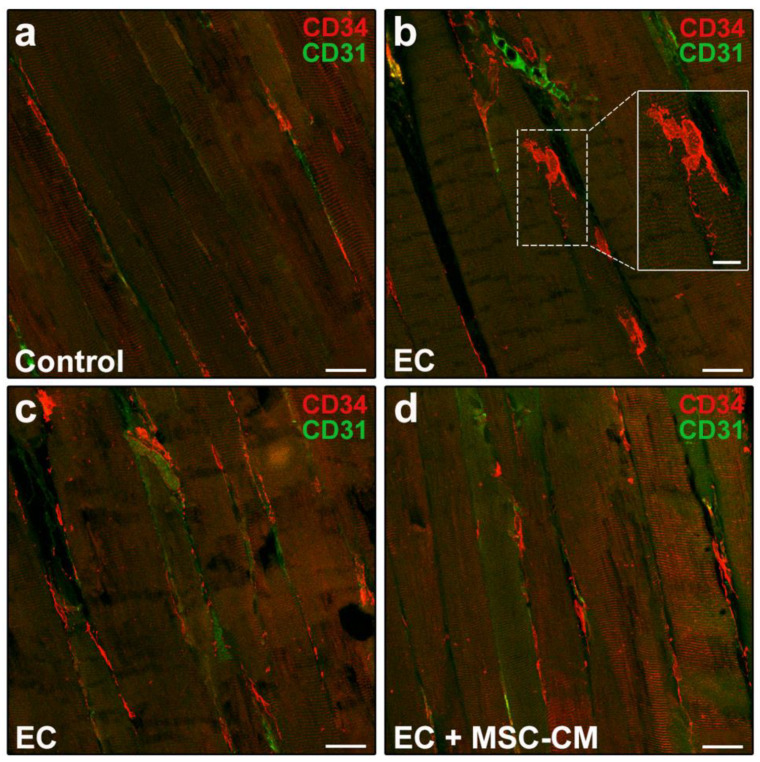
Morphological evaluation of telocytes in formalin-fixed and paraffin-embedded tissue sections from control EDL muscles, EDL muscles damaged by ex vivo forced eccentric contraction (EC) and EDL muscles damaged by EC in the presence of bone marrow-mesenchymal stromal cell (MSC) conditioned medium (EC + MSC-CM). (**a**–**d**) Representative confocal immunofluorescence images of muscle tissue sections immunostained with antibodies against CD34 (red) and the pan-endothelial cell marker CD31 (green). Telocytes are identified as CD34^+^/CD31^−^ stromal cells with distinctive moniliform cytoplasmic prolongations (telopodes), while vascular structures are CD31^+^. Higher magnification of the boxed area in (**b**) depicting a typical telocyte is shown in the inset. Images are representative of at least 3 sections from each of 4 control, 4 EC and 4 EC + MSC-CM muscles. Scale bars = 25 μm (**a**–**d**), 10 μm (inset in **b**).

According to relevant literature data and previous reports [8,35,36], telocytes were identified morphologically and immunophenotypically. These interstitial cells possess a small cell body abruptly giving rise to very long and thin moniliform cytoplasmic prolongations often with a sinuous course, called telopodes [35]. CD34 is considered the most reliable antigenic marker for in situ identification of telocytes by light microscopy and, hence, they are commonly referred to as telocytes/CD34^+^ stromal cells [36]. Given that blood vessel endothelial cells also express this marker, to unequivocally distinguish the two cell types, the simultaneous detection of CD34 and of the endothelial cell-specific CD31/platelet-endothelial cell adhesion molecule-1 is mandatory, particularly when blood vessel lumen is not clearly visible. Thus, immunophenotypically telocytes are referred to as CD34^+^/CD31^−^ interstitial cells, whereas blood vascular endothelial cells are CD34^+^/CD31^+^. As far as lymphatic endothelial cells are concerned, they are CD34^−^/CD31^+^ and, therefore, do not exhibit immunophenotypic overlapping with telocytes in CD34/CD31 double immunostained tissue sections [37]. Telocytes were found located in the interstitium of both control and EC-damaged muscles (Figure 4a–c).

In particular, in control muscles, telocytes appeared as spindle-shaped cells with a relatively small cell body giving rise to few telopodes, localized alongside the myofibers (Figure 4a). Of note, in EC-damaged muscles (Figure 4b,c), the network formed by telopodes appeared particularly extended nearby CD31^+^ vascular structures and even around the myofibers, almost as if wrapping them (CD34 red fluorescent intensity evaluated in merged red/green images including at least 10 myofibers, mean ± SEM in arbitrary units: control 41.08 ± 1.86, EC 80.69 ± 11.84, EC + MSC-CM 50.38 ± 1.74; *p* < 0.01 for EC vs. control, *p* < 0.05 for EC + MSC-CM vs. EC, *p* = 0.61 for EC + MSC-CM vs. control).

**Figure 5:**
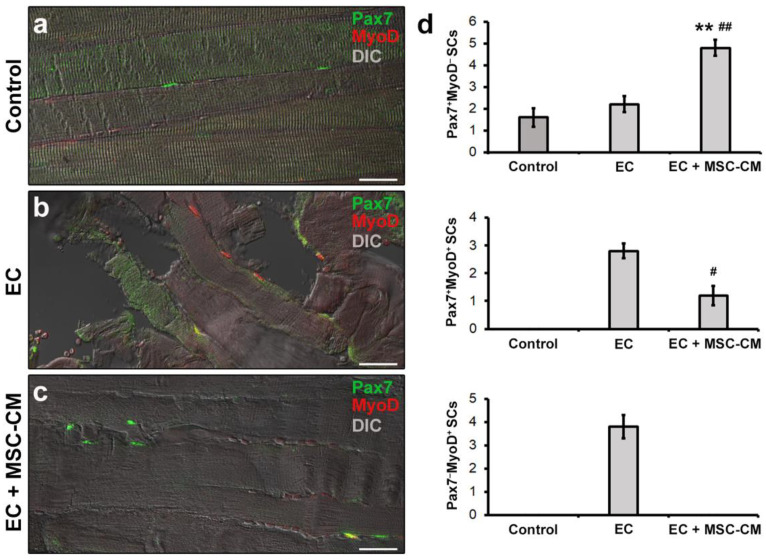
Morphological evaluation of satellite cells (SCs) in formalin-fixed and paraffin-embedded tissue sections from control EDL muscles, EDL muscles damaged by ex vivo forced eccentric contraction (EC) and EDL muscles damaged by EC in the presence of bone marrow-mesenchymal stromal cell (MSC) conditioned medium (EC + MSC-CM). (**a–c**) Representative superimposed confocal immunofluorescence and differential interference contrast (DIC, grey) images of muscle tissue sections immunostained with antibodies against the SC marker Pax7 (green) and the SC activation marker MyoD (red). Quiescent SCs or SCs likely contributing to replenish SC basal pool are identifiable as Pax7^+^/MyoD^−^ (green), tissue damage-activated SCs as Pax7^+^/MyoD^+^ (yellow), and SCs committed into the myogenic program as Pax7^−^/MyoD^+^ (red). Images are representative of at least 3 sections from each of 4 control, 4 EC and 4 EC + MSC-CM muscles. Scale bars = 25 μm. (**d**) Quantitative analysis of Pax7^+^/MyoD^−^, Pax7^+^/MyoD^+^ and Pax7^−^/MyoD^+^ SCs in the different experimental conditions. Values are mean ± SEM. ** *p* < 0.01 vs. control; # *p* < 0.05 and ## *p* < 0.01 vs. EC.

As far as SC analysis is concerned, tissue sections were double immunolabeled to simultaneously detect Pax7, regarded as the most reliable SC marker [38] and MyoD, the SC activation marker [39]. It is known that in healthy adult muscles Pax7^+^ satellite cells exist in a quiescent state. After tissue damage they become activated, co-express Pax7 and MyoD, and proliferate. Subsequently, these cells may adopt two alternative fates: most of them lose Pax7, maintain MyoD expression (Pax7^−^/MyoD^+^) and progress into the myogenic program required to form new myofibers; others maintain Pax7 and down-regulate MyoD (Pax7^+^/MyoD^−^) contributing to SC reservoir [39,40]. As expected, confocal microscopy assessment of control muscle samples revealed the presence of only few Pax7^+^/MyoD^−^ SCs typically juxtaposed to myofibers (Figure 5a,d).

In EC-damaged muscles, we detected few Pax7^+^/MyoD^−^ SCs not significantly different in number from control (*p* = 0.58) but, differently from control, the presence of activated Pax7^+^/MyoD^+^ and differentiating Pax7^−^/MyoD^+^ SCs that were undetectable in the control samples (Figure 5b,d).

Of interest, muscles subjected to EC in the presence of MSC-CM showed a reduced distribution of the interstitial network formed by telocytes and their telopodes as compared to EC (Figure 4d). Parallelly, in these samples we observed an increased number of Pax7^+^/MyoD^−^ SCs with respect to control and EC muscle (*p* < 0.01) and a decreased number of activated Pax7^+^/MyoD^+^ ones as compared to EC (*p* < 0.05) (Figure 5c,d). Differentiating Pax7^−^/MyoD^+^ SCs were undetectable.

**Figure 6:**
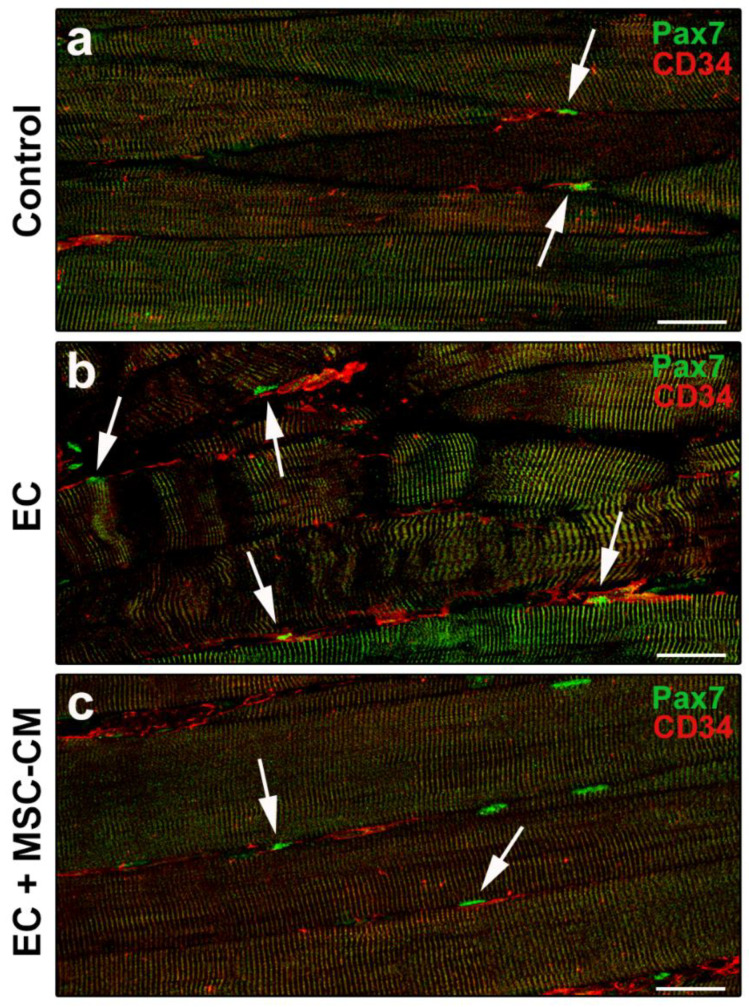
Morphological evaluation of telocytes and satellite cells (SCs) interaction in formalin-fixed and paraffin-embedded tissue sections from control EDL muscles, EDL muscles damaged by ex vivo forced eccentric contraction (EC) and EDL muscles damaged by EC in the presence of bone marrow-mesenchymal stromal cell (MSC) conditioned medium (EC + MSC-CM). (**a–c**) Representative confocal immunofluorescence images of muscle tissue sections immunostained with antibodies against Pax7 (green) and CD34 (red). Arrows indicate the presence of CD34^+^ telocytes in the close vicinity of Pax7^+^ SCs. Images are representative of at least 3 sections from each of 4 control, 4 EC and 4 EC + MSC-CM muscles. Scale bars = 25 μm.

The double immunolabeling for CD34 and Pax7 to simultaneously unravel telocytes and SCs allowed us to highlight the proximity of these two cell types, likely suggestive of a morpho-functional relationship (Figure 6a–c).

## 3. Discussion

In the present study, we have provided compelling experimental evidence of the capability of MSC secretome to exert a protective effect against EC-induced skeletal myofiber damage and to modulate the behavior of SCs that are key players of muscle tissue regeneration. Our findings contribute to further support the beneficial effects exerted by MSCs on skeletal muscle tissue. So far, MSCs have been the object of intense research in animal models aimed to the treatment of different skeletal muscle injuries or diseases raising many promises [2,13,14,15,16,17,18,19,20,21,25,28,29]. Indeed, the results of in vivo studies showed that these cells may facilitate the healing of injured muscles by promoting angiogenesis [13,19,25], increasing the number of regenerating myofibers [13,14,16,18,19,20,25,28,29], modulating the inflammatory response [19,30], reducing fibrotic response/scar formation [19,21,25] and improving muscle functional recovery [13,15,18,21,28].

In agreement with previous reports [18,22,23,24,25,27,28,29,30,41], our results suggest that paracrine mechanisms are mainly involved in the MSC beneficial effects related to the preservation of morpho-functional properties of skeletal myofibers during damage induction. In particular, we herein show that MSC secretome in the form of CM is effective in counteracting the EC-induced tissue structural damage, preventing myofiber apoptosis and upregulating AKT pro-survival factor expression [34]. Furthermore, the treatment with MSC-CM attenuated the functional tissue damage induced by EC, almost reverting the majority of the biophysical parameters toward control values and, thus, protecting the skeletal muscle fibers from irreversible deleterious events that affect the membrane functionality and excitability. As far as the voltage-dependent currents are concerned, EC provoked a reduction in the overall I_K_ size compared to control myofibers. This can be ascribed to a change in the opening status of K^+^ channels induced, at least in part, by the high-K^+^ solution used to provoke the forced EC. In fact, this solution tends unavoidably to depolarize the myofiber membrane, changing the Nernst potential for K^+^ ions and consequently the RMP value. Despite the subsequent relaxation in the MSC proliferation medium and the possibility of the myofiber to restore the normal RMP, the systematic repeating of the EC cycles in the two different bath solutions may induce a variation in the opening conformation of the different sarcolemnic voltage-dependent ion channels, which may also be influenced by the inactivation process. In this case, K^+^ efflux is hampered and this can justify the observed I_K_ reduction in EC-damaged myofibers. Notably, the presence of MSC-CM during the relaxation after EC cycles allowed the K^+^ channels to recover a good functionality only for voltage steps more positive than −20 mV. We can speculate that some factors present in the MSC-CM may trigger a signaling cascade of second messengers positively regulating only a precise set of ion channels that are critically activated around this voltage threshold. This kind of modulation should increase an intrinsic channel opening probability drastically reduced by the EC procedure. This hypothesis could justify the recovery of I_K_ current values in EC + MSC-CM toward physiological values, observed from −20 mV, and it is worth further investigation in future studies. Considering that K^+^ channels are usually regarded as majorly being responsible for RMP control, this observation agrees with the RMP measurements, where we did not actually observe significant recovery in EC + MSC-CM damaged myofibers compared to EC ones. Indeed, the RMP measured in these two conditions fall in a voltage range where I_K_ amplitude seems not to be significantly different. Since the similarity of I_K_ current amplitude in this voltage range is true also for control myofibers, whereas their RMP was significantly different from the damaged ones (EC in the absence or presence of MSC-CM), we must admit that the damage encompasses also a diverse target of proteins in causing RMP alteration. These can include, for instance, the membrane ATPases and/or a different family of ion channels, both voltage- and not voltage-dependent. Based on our results, we can reasonably suggest a role for not voltage-dependent (i.e., stretch operated channels) cation channels as further being responsible for the higher depolarization of RMP observed in EC-damaged myofibers. This idea is supported by the observation of the extremely reduced R_m_ measured in EC myofibers, which favors an enhanced basal ion flux that in turn reduces the negative charges distributed along the internal side of the sarcolemma. Concerning the observed induction of myofiber apoptosis, we may hypothesize that the sarcoplasmic Ca^2+^ ions increase, occurring during the EC damage as a main player. Different routes can determine its increment and contribute to the apoptotic scenario. For instance, calcium release from the endoplasmic reticulum can also induce inner mitochondrial pathway of apoptosis in a variety of cells [42]. As well, in skeletal muscle cells, T-type Ca^2+^ channels can induce endoplasmic reticulum Ca^2+^ disorder, contributing to mitochondrial-related apoptosis [43]. In this regard, the role of Ca^2 +^-release channel, ryanodine receptor (RyR) and inositol trisphosphate receptor (IP3R), other than T-type Ca^2+^ channels, have been extensively studied in the regulation of apoptosis in different preparations [44]. In our experimental model, during the several cycles of contractures, abundant Ca^2+^ ions are released in the sarcoplasm following the membrane depolarization, coming both from internal stores and from external medium to allow myofibrils activation and myofiber contraction. In normal conditions (control) the muscle can relax thanks to internal Ca^2+^ removal operated by specific pumps, located on the sarcoplasmic reticulum, namely SERCA: enduring internal Ca^2+^ homeostasis is an essential feature for the proper structure and physiological function of skeletal muscle and is critically required for maintaining homeostatic conditions and structural integrity [45]. However, the physiological equilibrium of Ca^2+^ ions can be altered during EC, thus providing extra-work for these structures and leads to an apoptotic condition. Different studies report a role both for RyR and for SERCA in apoptosis, showing a dysregulation of their function and or expression [42]. We tentatively suggest that these routes of Ca^2+^ ions regulation may not only be affected during the muscle damage, but may also be the outstanding target proteins of protective action of MSC-CM. Moreover, we may also hypothesize that many small molecules in the CM, which could help in changing the opening status of ion channels and other membrane proteins, may affect cytoskeletal organization and remodeling and, thus, cell membrane tension, stability and permeability. In fact, eccentric contraction is known to elicit a cytoskeletal remodeling response to limit the extent of the mechanical stress-induced damage, and it is also possible that actin cytoskeleton may transmit signals to the nucleus of the muscle cells through the activation of peculiar mechanical Ca^2+^ channels and biochemical processes leading to cell survival and protection [7,33]. Undoubtedly, this aspect deserves to be better explored in future investigations.

Another interesting finding of our study is the capability of MSC-CM to influence even the behavior of SCs and telocytes, two cell types involved in skeletal muscle tissue repair/regeneration [8]. In particular, MSC-CM, while protecting fully developed myofibers from damage, prevented the EC-induced myogenic commitment of SCs and increased the number of SCs likely replenishing the basal pool, recruitable in the case of muscle tissue re-injury. These observations agree with our previous in vitro studies focused on the investigation of the cross-talk among MSCs and the muscle progenitor cells unveiling the capability of MSCs to stimulate skeletal myoblast and SC proliferation [23,24,27,41]. In addition, in line with our observation, Mitchell and co-workers [46] recently demonstrated a similar response of SCs from cardiotoxin damaged muscles after treatment with the secretome of adipose-derived stem cells, which share many biological properties with adult mesenchymal stem cells from the stem niche of different tissues including bone marrow [47]. As far telocytes are concerned, by means of their distinctive telopodes, these peculiar stromal cells have been reported to form an interstitial network that is more extended in EC-damaged muscle compared to control undamaged muscle [8]. Interestingly, when EC was performed in the presence of MSC-CM, the stromal network of telocytes/telopodes resulted in being less extended, similar to the control. Of note in all experimental conditions, telocytes were observed in the close vicinity of SCs likely contacting them. These cells may then be considered an essential modulator for SC functionality, corroborating telocytes as “nursing” cells within the SC niche [8,48] as they have been proposed in the stem cell niche of different tissues [49]. Experiments are ongoing in our laboratory to deepen the morpho-functional relationship between telocytes and muscle progenitor cells in physiological and pathological conditions.

The bioactive factors contained in the CM mediating the beneficial effects of MSCs on skeletal muscle in our model need to be clearly identified. However, we may speculate that the bioactive lipid sphingosine 1-phosphate (S1P) and the vascular endothelial growth factor (VEGF) may represent two potential effector molecules likely acting synergistically. This idea is based on: (i) our previous demonstration of the presence of both S1P and VEGF in the murine MSC-CM employed in the present study [23,26,41,50]; (ii) the S1P ability to attenuate the EC-induced tissue damage, protecting skeletal myofibers from apoptosis, preserving SC viability [33], and exerting multiple beneficial effect on skeletal muscle [7]; (iii) the well-established role of VEGF in adult skeletal muscle physiology as well as its action on SCs [41,51,52]; and (iv) the well-demonstrated AKT activation induced by S1P and VEGF [53,54,55].

In conclusion, the present data contribute to support the beneficial effects of MSCs on muscle damage and may have important implications in the optimization of therapeutic strategies to manage skeletal muscle damage, likely promoting skeletal muscle repair/regeneration. However, we are aware of the limitations of our study that are mainly related to the ex vivo model of muscle damage, not allowing us to evaluate the complete tissue regenerative process, the lack of a full characterization of the releasing profile of the factors contained in the MSC secretome and, therefore, the lack of a clear identification of the mechanisms underpinning its protective action. Nevertheless, our novel findings strengthen the notion that the use of MSC-CM may represent a valid alternative to cell therapy in muscle regenerative medicine, avoiding drawbacks related to cell transplantation procedures including, among others, low cell survival in the host tissue or cardiovascular risk [28,29,30,56]. Moreover, the administration of a well-characterized secretome whose active components and their specific effects are clearly identified may lead to more predictable outcomes and a more controlled treatment. Indeed, this seems extremely important in a therapeutic setting, especially when considering that MSCs may change their functionality in terms of differentiative potential and secretory ability in response to stimuli from the recipient microenvironment with possible undesirable effects [57]. As always, future bench to bedside translation must be considered with extreme caution.

## 4. Materials and Methods

### 4.1. MSC Culture and CM Harvesting

For the present experimentation, thawed MSCs previously collected from femora and tibiae bone marrow of male C2F1 mice, expanded in vitro, characterized immunophenotypically and morphologically, and cryopreserved in a solution containing 10% dimethyl sulfoxide and 90% heat-inactivated (at 56 °C for 30 min) fetal bovine serum (FBS, Sigma-Aldrich, St. Louis, MO, USA), in a liquid nitrogen tank [27] were used. After proper thawing, the cells (2 × 10^6^) were cultured on 35 mm dishes in their specific proliferation medium (1.5 mL) containing DMEM supplemented with 20% FBS, and 1% penicillin/streptomycin (Sigma-Aldrich). After 24 h, the culture medium (i.e., MSC-CM) was harvested and used during EC experiments.

### 4.2. Ex Vivo Murine Model of Muscle Damage

Ex vivo experiments were achieved on EDL muscles isolated from 10 male Swiss mice (25–30 g) intramuscularly anesthetized with Xylazine (5 mg/kg; Sigma-Aldrich) Lidocaine (2% *w*/*v*; Sigma-Aldrich) and Zoletil (Virbac S.r.l., Milan, Italy). A suitable depth of anesthesia was kept so that the mice were insensitive to tactile stimulation. Once excised by cutting the tendons, EDL muscles (*n* = 7) were injured by subjecting it to forced EC in isometric condition, essentially according to the previously published procedure with minor modifications [8,33]. In brief, the EDL muscle was injured through stretching (stretch extent ~20% of the muscle resting length) and exposure to a series of contractures (ten cycles of 1 min each) in the following solution with a high K^+^ concentration: 150 mM K-glutamate, 2.0 mM MgCl_2_, 10 mM KOH, 10 mM TES and 1.0 mM K_2_-EGTA (Sigma-Aldrich). After each cycle of EC, the muscle relaxed for 4 min in MSC proliferation medium. Alternatively, a group of muscles (*n* = 7) was subjected to stretching and exposure to a series of contractures (ten cycles of 1 min each) and relaxed after each cycle for 4 min in MSC-CM (EC + MSC-CM). Excised muscles not subjected to EC were used as control (*n* = 6). After EDL muscle removal, the anaesthetized mice were rapidly sacrificed by cervical dislocation.

Mice were housed in the Laboratory Animal Facility (CeSAL, Centro Stabulazione Animali da Laboratorio, University of Florence, Florence, Italy), maintained at 23  ±  1  °C with a 12  h light/dark cycle, fed with standard laboratory diet and tap water ad libitum. All of the animal handlings were carried out in agreement with the Directive 2010/63/EU of the European Parliament and of the European Union council (22 September 2010) on the protection of animals used for scientific purposes. 

### 4.3. Light Microscopy

Control (*n* = 4), EC-damaged (EC, *n* = 4) and EC + MSC-CM (*n* = 4) muscle samples were processed for paraffin embedding as previously reported [8]. Deparaffinized tissue sections (5 μm thick) were routinely stained with H&E. Observations were performed with a light microscope (Leica DM4000 B) furnished of a digital color camera (DFC310 FX 1.4-megapixel) and the software application suite LAS V3.8 (Leica Microsystems, Mannheim, Germany). The number of myofibers with the typical morphological signs of cell damage was evaluated by two independent observers and reported as percentage of the total number of myofibers per microscopic field (×40).

### 4.4. Confocal Laser Scanning Microscopy

Paraffin-embedded muscle tissue sections (5 µm thick) were processed for confocal laser scanning microscopy analysis as reported previously [8]. The fluorescent dye PI (1:100, for 2 min at room temperature, Molecular Probes, Eugene, OR, USA) was used to label nuclei. For confocal immunofluorescence analyses, the following primary antibodies were used (4 °C, overnight): mouse monoclonal anti-Cyt c (1:200, catalog no. 556432; BD Biosciences, San Jose, CA ), rabbit polyclonal anti-cleaved caspase-3 (1:100, catalog no. #9664; Cell Signaling Technology, Danvers, MA, USA), rabbit polyclonal anti-AKT (1:100, catalog no. #4691; Cell Signaling Technology), rabbit monoclonal anti-p-AKT (1:100, catalog no. #4060; Cell Signaling Technology), rabbit monoclonal anti-CD34 (1:50; catalog no. ab81289; Abcam, Cambridge, UK), rat monoclonal anti-CD31 (1:100; catalog no. ab7388; Abcam), mouse monoclonal anti-Pax7 (1:100; catalog no. sc-81648; Santa Cruz Biotechnology, Santa Cruz, CA, USA), rabbit polyclonal anti-MyoD (1:50; catalog no. sc-760, Santa Cruz Biotechnology). Muscle samples immunolabeled for Cyt c detection were incubated with Alexa Fluor ^TM^ 488-conjugated wheat germ agglutinin (WGA, 1:100; Thermo Fisher Scientific, Waltham, MA, USA) solution for 10 min according to the manufacturer’s instructions, to label cell membrane. The following specific secondary antibodies (1 h, room temperature) were employed to reveal primary ones: anti-rabbit or anti-mouse or anti-rat Alexa Fluor 488-conjugated IgG (1:200; catalog no. A11034, A11001, A11006, Molecular Probes) or anti-rabbit or anti-mouse 568-conjugated IgG (1:200; catalog no. A11011, A11036, Molecular Probes). Negative controls were obtained by substituting primary antibodies with non-immune serum and the secondary antibodies cross reactivity was assessed by omitting primary antibodies. Observations were performed by using a confocal Leica TCS SP5 microscope (Leica Microsystems) as reported previously [8].

In the EC and EC + MSC-CM muscle tissue samples, the number of myofibers showing levels of Cyt c expression higher than those of myofibers in the control group was counted on WGA/Cyt c digitized images (at least 10) and expressed as a percentage of total myofibers. The Cyt c fluorescent signal intensity relative to control myofibers was measured by using ImageJ 1.49v software (NIH, https://imagej.nih.gov/ij/, accessed on 22 February 2021) and considered as a threshold to subtract to the Cyt c fluorescent signal intensity relative to EC and EC + MSC-CM samples. The number of myofibers expressing cleaved-caspase-3 was evaluated on at least 10 superimposed DIC/fluorescence images (200 μm^2^ optical fields, ×63) by two independent operators (the count numbers were then averaged) and expressed as a percentage of the total myofibers. Densitometric analysis of AKT fluorescent signal intensity was performed on digitized images (at least 10) by using ImageJ 1.49v software in 30 regions of interest (100 μm^2^) for each image. The number of myofibers expressing cleaved-caspase-3 was evaluated on at least 10 superimposed DIC/fluorescence images (200 μm^2^ optical fields, ×63). For each tissue section, the intensity of CD34 fluorescent signal not colocalized with the green one was evaluated on at least 10 red (CD34)/green (CD31) merged images (200 μm^2^ optical fields, ×63 Leica TCS SP5 microscope) including at least 10 myofibers. The number of SCs was evaluated on 5 random 200 μm^2^ optical square fields ×63, Leica TCS SP5 microscope) containing at least 10 myofibers in each tissue section by two independent observers; the count numbers were then averaged. At least three different tissue sections from each EDL muscle were analyzed.

### 4.5. Electrophysiological Analysis

The electrophysiological records were attained on skeletal myofibers from control muscles (*n* = 2), EC- muscles (*n* = 3) or EC + MSC-CM muscles (*n* = 3) essentially as described in previous papers [8,33]. The muscle was continuously superfused during the experiments at a rate of 1.8 mL min^−1^ (Pump 33, Harvard Apparatus LTD, Edenbridge, Kent, UK) in the experimental chamber containing the Ringer-Krebs solution (mM): NaCl 120, KCl 5, CaCl_2_ 2, MgCl_2_ 1, HEPES 5.5, and D-glucose 1 (Sigma-Aldrich), at room temperature (22 °C). RMP was recorded in the current clamp mode (stimulus waveform I = 0 pA) by inserting a microelectrode into a single myofiber of the muscle bathed in physiological solution. The microelectrodes were pulled by a micropipette vertical puller (Narishige PC-10; Narishige International Inc., East Meadow, NY, USA) from borosilicate glass capillaries (GC 100-7.5; Harvard Apparatus LTD) and were filled with an internal filling pipette solution containing (mM): KCl 130, NaH_2_PO_4_ 10, CaCl_2_ 0.2, EGTA 1, MgATP 5 and HEPES/KOH 10 (pH 7.2) (Sigma-Aldrich). The tip resistance was about 60 MΩ. The junction potential of the electrode was measured prior to make the patch, and then it was subtracted from the recorded membrane potential. The membrane passive properties of the myofibers (C_m_ and R_m_) as well as I_K_ were measured in voltage-clamp mode. To estimate the membrane passive properties two 75-ms long voltage step pulses to −80 and −60 mV were usually applied, starting from a holding potential (HP) of −70 mV.

To evoke the ionic transmembrane currents, we applied 1-s long voltage pulses ranging from −80 to +50 mV in 10-mV increments (HP = −60 mV). The P/4 procedure allowed us to cancel on line any leakage and voltage-independent ionic currents from the records. The current amplitude (I) was normalized to C_m_ (in pA/pF) to properly compare the currents recorded from different myofibers. The I/C_m_ values represent the current density (in pA/pF). The digital-to-analog and analog-to-digital conversions were carried out by using a Digidata 1200 interface (Axon Instruments, Burlingame, CA, USA). pCLAMP programs (version 6.02 and 9.0, Axon Instruments) were used for stimulation, recordings and data acquisition. The condition of the myofibers was constantly checked by monitoring the RMP and R_m_. Myofibers remained stable for about 150–240 min.

### 4.6. Statistical Analysis

Electrophysiological data are expressed as means ± standard deviation (SD). Comparison between more than two groups of samples was made by one-way ANOVA and Bonferroni’s post hoc test. Image/morphological data are reported as mean ± standard error of the mean (SEM) of at least three independent experiments performed in triplicates. One-way ANOVA with post-hoc Tukey HSD were used to perform the statistical analysis of differences among the experimental groups. Statistical significance was set at *p* < 0.05.

## Data Availability

All relevant data are included within the manuscript.
